# The unlikely legalization of medical cannabis in Albania: a case study

**DOI:** 10.18502/jmehm.v12i10.1447

**Published:** 2018-08-27

**Authors:** Gentian Vyshka

**Affiliations:** *Faculty of Medicine, University of Medicine, Tirana, Albania.*

**Keywords:** Medical ethics, Cannabis, Legalization, Tetra-hydro-cannabinol (THC), Policymaking

## Abstract

Cannabis abuse has been an issue of major concern for the Albanian society in recent years, following the wide illegal planting of the species. Legal lacunas, weaknesses from the drug-controlling agencies, and the easiness of harvesting *Cannabis sativa *plants have contributed to the creation of a general negative opinion toward a potential use of its active principles for medical purposes. Professionals of the field and policymakers are largely focused on harm reduction activities, thus bypassing the option of legalizing cannabinoids for clinical conditions that might find relief in their use.

The discussion of a case suffering from postherpetic neuralgia shows how this negative opinion is depriving Albanian patients from an otherwise helpful pharmacological option whose efficacy has been confirmed by an important bulk of research. Uncovering the roots of this misperception will help address the issue. Clinicians with expertise need to have their say in the debate, since for the majority of countries where medicinal cannabis is legalized, this was due to consumer-led initiatives. Ensuring patients’ freedom as implied within the principle of autonomy is also a sound ethical support of such legalization.

## Introduction

Cannabis and marijuana have become an issue of major concern in Albania in recent years, mainly due to an unprecedented wave of illegal planting and trafficking. A variety of reasons have led to this phenomenon, including economic hardship, legal lacunas and law enforcement weaknesses, the high quality of the plant in terms of THC (tetra-hydro-cannabinol) concentrations, and the easiness of its harvest (1). 

This situation has rendered very unlikely the option of legalizing medical cannabis, at least in the near future. Not only is the public perception of cannabis strongly deformed, but also prejudice and ignorance among policy-makers and professional staff is seriously hampering its legalization. Beyond the absent discussions about the clinical adequacy or lack of efficacy of the THC and cannabinoids in certain medical conditions, Albanian politicians, professionals and laypersons are conjointly focusing on harm reduction approaches (2, 3). 

This is obviously related to the fact that cannabis is the most widely abused agent after alcohol, according to recently published data. Thus, ethanol apart, it might account for more than 80% of all cases of drug abuse, including illegal agents such as heroin and cocaine, or sedatives licensed for medical purposes (with benzodiazepines on the top of the list) (4). 

## Case description

An octogenarian with severe post-herpetic neuralgia, unresponsive to all licensed drugs used in Albania for pain, neuropathy and chronic painful conditions, was desperately seeking relief. In spite of consultancies and referrals in pain centers, the aching and suffering was relentless, especially at night. Conventional antalgics (non-steroidal anti-inflammatory drugs), tramadol and oxycodone were sequentially administered with very little success, if at all.

After several sessions, visits and examinations, the treating clinician lost contact with the patient. A few weeks later he met by chance the old man’s son, who told the doctor that each evening he was giving his father some drops of handmade hashish oil whose concentration he ignored, but that worked pretty well.

In this case, cannabis was not being used under professional supervision, but that does not necessarily mean the treatment was illogical from the medical point of view. The patient’s son was obviously breaking the common law, even facing arrest if caught with whichever quantity of hashish. Yet the drug was achieving what had seemed impossible: it was alleviating the unbearable pain of his father.

## Discussion

Some moral dilemmas might be formulated in relation to this case. The family physician was no more in the position of a treating doctor, since he was unaware of the treatment. The post-factum searching of his opinion could not contradict the efficacy of the drug, especially when all other previous options had failed. Turning a blind eye would probably exculpate the doctor from not denouncing a situation of illegal use of prohibited drugs, which seems not the case as long as that happened out of his sight. 

Questioning the morality and the moral basis of offering a therapeutic option to aching patients in painful conditions – in this case cannabis – is an issue that has raised discussions and arguments even among judicial authorities and scholars (5, 6). 

Demonizing cannabis *in toto* and upholding an aprioristic position of exclusion is mainly due to a defective perception of the plant. This misperception is obviously depriving Albanian patients from an otherwise helpful pharmacological option, with the general public equalizing its usage with crime and antisocial behavior in the worst of cases, or exhibiting a sense of phobia in others. Politics has largely prevailed over medical reasons, and it seems this is not a local or a localized phenomenon (7). Weak and flawed political discussions have hardly helped the progress toward a probable legalization for medical purposes, and after an initial acme during the years 2014 - 2015, the issue has been almost entirely pushed aside. At that time there were some Albanian members of the parliament suggesting a mitigation of legal penalties for cannabis abusers, together with its legalization for medical purposes. With the society having other priorities, and the general public ignoring the quintessence of the discussion, oblivion prevailed in the midst of an ever flourishing illegal harvesting and consumption. Uncovering the roots of this misperception will help address the issue, and redress the prejudicial elimination of medical cannabis from being available on the market. 

Very few attempts have been made to uncover the medical values of the plant to the Albanian public, whose perception of cannabis consumption remains strongly impregnated with fear and a feeling of doing something wrong, even if not caught and prosecuted. A big deal of this misperception relies on the terminology. In fact, hashish entered the everyday Albanian language not directly from the Arab language, but rather during the historical period when the country was part of the Ottoman Empire. The misspelling of *hashish* (from Arabic “grass”) as *hashash* (the later usage of Turkish *haşhaş*, which means “opium”) has largely served to increase confusion, and made laypersons consider cannabis as strong, dangerous and addictive as heroin itself. On the other hand, Latin influences probably have not spared Albanian semantics: for most of the Latin-derived languages, *hashish* may have served as an origin to the word *assassin*, although this still might be an etymological speculation. Some authors suggest the term to stem from the order of the *haschischins*, founded in the sixth century A.D., whose members consumed cannabis deliberately during combat preparations (8). 

Since the chemical extraction of its active principle (THC, Tetra-hydro-cannabinol) more than half a century before, attempts toward convincing policymakers in favor of legalizing cannabis for medical purposes have not been systematic and unfortunately not always successful. As one of the THC discoverers and an author on the subject, Mechoulam has reported his experiences and suggested obvious advantages that produces medical cannabis for patients in need, as an agent already legalized in scores of countries (2, 9). Apart from having intrinsic antinociceptive effects, cannabis-derived byproducts (THC and other cannabinoids) might be useful for their antalgic properties, especially when aiming at lowering the dosage of opiates, if not substituting these completely in selected conditions (10, 11). 

The major drawback of all these debates is that clinicians, i.e. the authorities that will prescribe cannabis once medically legalized, have been largely silent (12). During an accurate analysis of the issue, Newton-Howes and McBride conclude that medicinal cannabis has mostly been a consumer-led initiative, and that the socio-political prejudices of the day contribute more toward its prohibition, rather than focusing on the real harms (12).

## Conclusion

In an important, delicate issue such as legalizing cannabis for medical purposes, physicians and treating clinicians need to have the final say. Clearly, the legal status of cannabis as a drug of abuse must remain separated from the medicinal framework (12). Large-scale and multicenter clinical trials that will convince policymakers to opt for marketing medical cannabis are difficult to implement as long as prohibition stands strong. Even the contrary perspective, i.e. the view that the prohibition of cannabis can be based on medical reasons, is erroneous (13). To make decisions more simple, comprehensive and logical, one should remember that freedom of individuals as implied within the principle of self-determination or autonomy seems a sound ethical reason to allow the use of medical cannabis (13). 

Ethics and the slippery-slope arguments, for instance policymakers’ fear that cannabis legalization will serve as a gateway for stronger and earlier addiction disorders, remain elusive (14, 15). Yet this position, so far, is hampering a logical and medically-oriented *ad hoc* legislation in Albania, when some countries have already advanced in this direction and enacted it accordingly (5, 6, 16).

## Citation to this article:

Vyshka G. The unlikely legalization of medical cannabis in Albania: a case study. J Med Ethics Hist. 2019; 12: 10.
